# Cytopenias in context: ethnoracial hematological trends among HIV-infected Africans and Russians: a review

**DOI:** 10.1097/MS9.0000000000004520

**Published:** 2025-12-16

**Authors:** Emmanuel Ifeanyi Obeagu, Olga Goryavheva Goryavheva, Mikhail Anatolyevich Zubarev

**Affiliations:** a Department of Biomedical and Laboratory Science, Africa University, Mutare, Zimbabwe; b Internal Disease, Perm State Medical University Named After Academician E.A. Wagner, Russia

**Keywords:** cytopenias, ethnoracial variability, hematology, HIV, immune suppression

## Abstract

Cytopenias – particularly anemia, leukopenia, and thrombocytopenia – are common hematological manifestations among individuals living with human immunodeficiency virus (HIV) and serve as critical markers of disease severity, progression, and treatment response. These hematologic complications often vary across populations due to a complex interplay of genetic, environmental, nutritional, and socioeconomic factors. Ethnoracial diversity significantly influences the expression and outcomes of these cytopenias, necessitating population-specific investigations to guide accurate diagnosis and effective clinical management. In this review, we explore and contrast the hematological profiles of HIV-infected individuals of African and Russian descent, highlighting key differences in prevalence patterns, underlying mechanisms, and associated comorbidities. African populations frequently exhibit higher rates of anemia and benign ethnic neutropenia, which can obscure diagnostic clarity. Meanwhile, Russian populations face unique challenges, including alcohol-induced marrow suppression and coinfections such as hepatitis C, which exacerbate leukopenia and thrombocytopenia. These disparities are further compounded by differences in healthcare access, nutritional status, and the timing of HIV diagnosis and treatment initiation.

## Introduction

Hematological abnormalities are among the earliest and most persistent complications observed in individuals living with human immunodeficiency virus (HIV). These abnormalities, collectively referred to as cytopenias, include anemia, leukopenia, and thrombocytopenia. They are not only markers of advanced disease and immune suppression but also contribute significantly to morbidity and mortality. Cytopenias compromise host immunity, predispose individuals to opportunistic infections, and interfere with antiretroviral therapy (ART) adherence and effectiveness. Despite the global burden of HIV, the hematological responses to the virus and its treatment are not uniform across populations^[1–5]^. Ethnoracial diversity plays a critical role in determining the baseline hematological parameters and the manner in which HIV-related cytopenias manifest. Factors such as genetic polymorphisms, environmental exposures, nutrition, socioeconomic status, and healthcare infrastructure contribute to these differences. African and Russian populations offer a particularly compelling comparison due to their distinct genetic backgrounds, geographic conditions, and divergent public health responses to HIV. A closer examination of the hematological trends within these two groups can provide important insights for targeted clinical management^[6–8]^. In African populations, cytopenias – particularly anemia and neutropenia – are highly prevalent among HIV-positive individuals. This is often compounded by late-stage diagnosis, limited access to ART, co-existing infections such as malaria and tuberculosis, and widespread nutritional deficiencies. Furthermore, benign ethnic neutropenia (BEN), a well-documented condition in individuals of African descent, can complicate the interpretation of leukocyte counts and delay timely intervention. These conditions collectively influence disease prognosis and treatment outcomes^[9,10]^.

**HIGHLIGHTS**

- Ethnoracial variations influence baseline hematologic values, affecting cytopenia diagnosis accuracy.
- Human immunodeficiency virus (HIV)-infected Africans show higher anemia prevalence than Russians.
- Neutropenia is more common among Africans due to genetic predispositions.
- Socioeconomic and nutritional disparities exacerbate cytopenias in African cohorts.
- Tailored hematologic reference ranges are vital for accurate HIV management across diverse populations.

Russian populations, while experiencing different sociopolitical and environmental pressures, exhibit a distinct profile of hematological complications. High rates of alcohol consumption, hepatitis B and C coinfections, and intravenous drug use influence the prevalence and severity of cytopenias in this population. Thrombocytopenia, in particular, is often associated with liver dysfunction and immune-mediated platelet destruction. Additionally, late presentation to healthcare facilities and stigmatization around HIV contribute to poor management of hematologic abnormalities^[11–13]^. The pathophysiology of HIV-related cytopenias remains complex and multifactorial. Direct viral invasion of the bone marrow, immune-mediated destruction of blood cells, chronic inflammation, ART toxicity, and coinfections all contribute to these abnormalities. However, the extent to which these factors affect individuals can vary significantly between ethnoracial groups. For example, zidovudine-induced anemia may have a greater impact on nutritionally compromised African patients compared to their Russian counterparts with better dietary support systems^[14,15]^. Moreover, differences in laboratory reference ranges across populations further complicate diagnosis and treatment. Most hematologic reference values used in clinical practice are derived from Western populations, which may not accurately reflect the baseline parameters in Africans or Russians. Without ethnically calibrated diagnostic criteria, clinicians may overlook significant hematological changes or misinterpret benign findings as pathological, leading to unnecessary ART changes or additional diagnostic testing.

**Figure 1.**
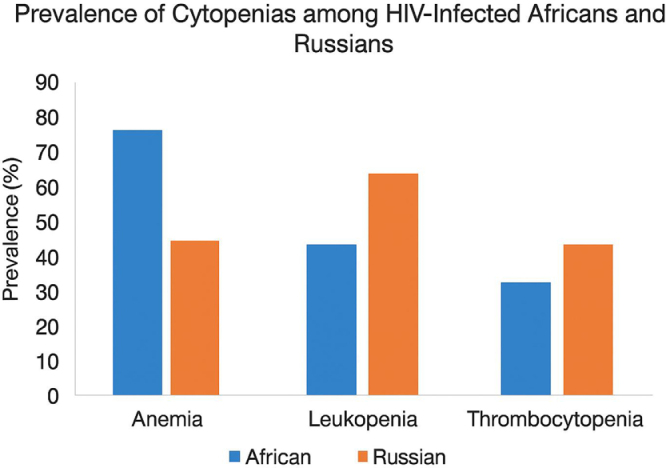
Prevalence of cytopenias among HIV-infected Africans and Russians.

Coinfection with hepatitis B virus (HBV) and hepatitis C virus (HCV) is a significant concern among people living with HIV (PLHIV) due to shared transmission routes and synergistic impacts on liver disease progression and treatment outcomes. In sub-Saharan Africa, HBV coinfection among HIV-infected individuals ranges from 5% to 20%, with regional variations reflecting differences in endemicity, vaccination coverage, and vertical transmission rates. HCV coinfection is comparatively less common in Africa, with prevalence estimates generally below 5%, although higher rates are observed among high-risk populations such as intravenous drug users. In contrast, HIV/HCV coinfection is substantially more prevalent in Russia, affecting approximately 25%–30% of PLHIV, largely driven by intravenous drug use and historical gaps in harm reduction programs, while HIV/HBV coinfection occurs in 3%–10% of cases. These coinfections complicate HIV management by increasing the risk of hepatotoxicity during ART, accelerating liver fibrosis, and altering immune recovery, underscoring the need for integrated screening and management strategies in both African and Russian settings (Fig. 1)^[14,15]^.

## Aim

The aim of this review is to explore the ethnoracial variations in the presentation and management of HIV-associated cytopenias among African and Russian populations.

## Methods

This narrative review was conducted following a structured literature search to synthesize current knowledge on ethnoracial hematological trends among HIV-infected Africans and Russians. A comprehensive search of PubMed, Scopus, Web of Science, and Google Scholar databases was performed for articles published between 2000 and 2025. Keywords included combinations of “HIV,” “cytopenias,” “anemia,” “leukopenia,” “thrombocytopenia,” “pancytopenia,” “Africa,” “Russia,” “ethnoracial,” and “hematological trends”.

A total of 142 articles were initially identified. After screening titles and abstracts for relevance, 94 full-text articles were selected for inclusion based on the following criteria: (i) studies reporting hematological parameters or cytopenias in HIV-infected adults, (ii) studies including African or Russian populations, and (iii) observational studies, clinical trials, or systematic reviews/meta-analyses providing quantitative or qualitative data relevant to cytopenias. Articles were excluded if they were non-English, focused solely on pediatric populations without adult data, or did not provide specific hematological outcomes.

Data extracted included study design, sample size, population characteristics, prevalence and types of cytopenias, coinfections (HBV/HCV/TB), ART regimens, and relevant genetic or environmental factors. Evidence quality was assessed narratively, with emphasis on higher-level evidence such as meta-analyses and cohort studies. Comparative analyses between African and Russian populations were emphasized where data were available. All abbreviations were defined at first use and applied consistently throughout the review.

### Overview of HIV-associated cytopenias

HIV infection is frequently associated with hematological abnormalities that reflect disease progression and immune status. Among the most prevalent of these are cytopenias – conditions characterized by reductions in one or more cellular components of blood. The primary forms of cytopenias in the context of HIV include anemia, leukopenia (especially neutropenia and lymphopenia), and thrombocytopenia. These cytopenias may occur independently or concurrently and are often multifactorial in origin, arising from a combination of direct viral effects, opportunistic infections, nutritional deficiencies, and antiretroviral or prophylactic drug toxicity^[16–18]^.

### Anemia

Anemia is the most common hematological abnormality among PLHIV and can present at any stage of infection, though it becomes more prevalent and severe as the disease advances. Its etiology is multifaceted, including bone marrow suppression due to HIV itself, chronic inflammation, opportunistic infections such as *Mycobacterium tuberculosis* and Parvovirus B19, and the use of myelosuppressive medications like zidovudine. Nutritional deficiencies, particularly of iron, folate, and vitamin B12, are also common contributors, especially in resource-limited settings. Anemia in HIV is often normocytic and normochromic, indicative of anemia of chronic disease, but microcytic or macrocytic patterns may also be observed depending on underlying causes^[5,19–21]^.

### Leukopenia

Leukopenia, particularly neutropenia and lymphopenia, is another frequent hematologic complication of HIV. Lymphopenia, especially a decline in cluster of differentiation 4 (CD4)+ T cells, is a hallmark of HIV progression and serves as a key marker for clinical staging and therapeutic decision-making. Neutropenia may arise from HIV-related bone marrow suppression, medication toxicity (notably from ganciclovir, cotrimoxazole, or zidovudine), and coinfections such as cytomegalovirus. In individuals of African descent, BEN may further complicate clinical interpretation. Severe leukopenia increases the risk of opportunistic infections and may necessitate treatment adjustments or granulocyte colony-stimulating factor (G-CSF) administration^[17,22–24]^.

### Thrombocytopenia

Thrombocytopenia in HIV-infected individuals can occur in both early and late stages of the disease. The mechanisms include immune-mediated platelet destruction, similar to idiopathic thrombocytopenic purpura (ITP), and reduced platelet production due to megakaryocyte suppression by the virus or coexisting marrow infiltration. Additionally, liver dysfunction from hepatitis coinfection – common in some populations, such as Russians – can impair thrombopoietin production and increase platelet sequestration in the spleen. Clinically, thrombocytopenia may manifest as mucocutaneous bleeding or petechiae, but it is often asymptomatic and detected only through laboratory testing. Severe thrombocytopenia may require corticosteroid therapy, intravenous immunoglobulin (IVIG), or ART modification^[18,25–27]^.

### Ethnoracial hematological baselines and variability

The interpretation of hematological abnormalities in HIV-infected individuals must be contextualized within the framework of ethnoracial variability in baseline hematologic parameters. It is well established that genetic, geographic, environmental, and socioeconomic factors contribute to population-specific differences in hematologic indices. These differences have profound implications for the clinical diagnosis and monitoring of cytopenias in PLHIV, particularly among African and Russian populations^[28,29]^.

### African populations

In individuals of African descent, baseline hematologic values often differ from those of Caucasian or European populations. One of the most widely documented differences is the lower baseline neutrophil count, commonly referred to as BEN. This condition, characterized by chronically reduced absolute neutrophil counts without an increased risk of infection, is believed to result from the Duffy-null polymorphism and other genetic variants affecting granulopoiesis. In HIV-infected African populations, this baseline neutropenia can confound clinical evaluation, making it difficult to distinguish pathological neutropenia due to HIV progression or drug toxicity from benign ethnic variation. Anemia is also more prevalent and often more severe among African HIV-positive individuals. This may be attributed not only to the disease itself and ART-related bone marrow suppression but also to high rates of iron deficiency, malaria, helminthic infections, and poor dietary intake of essential micronutrients such as folate and vitamin B12. Moreover, the late diagnosis and initiation of ART – often seen in sub-Saharan African settings – predispose patients to more advanced disease with severe hematologic derangements at presentation. These trends necessitate the use of population-specific reference ranges and a high index of clinical suspicion when managing cytopenias in African patients^[28,30–33]^.

Emerging evidence suggests that other genetic variants may also modulate granulopoiesis and contribute to BEN, including CXCR2 gene variants, which influence neutrophil chemotaxis and maturation, and polymorphisms in cytokine and immune regulatory genes. In HIV-infected populations, BEN may complicate the interpretation of neutropenia, as low neutrophil counts may not necessarily indicate immunosuppression or ART toxicity. Recognizing the interplay of these genetic factors is essential for accurate diagnosis, avoiding unnecessary treatment modifications, and understanding ethnoracial hematologic patterns in HIV care[33].

### Russian populations

Conversely, the hematologic baseline parameters in Russian populations align more closely with Western European reference values, though unique challenges exist. Factors such as alcohol abuse, hepatitis C coinfection, and intravenous drug use have emerged as significant contributors to hematologic abnormalities in Russian individuals living with HIV. Alcohol-related marrow suppression can contribute to pancytopenia, while chronic liver disease impairs thrombopoietin synthesis and increases splenic sequestration, leading to thrombocytopenia. Additionally, parenteral drug use and inadequate nutritional intake further exacerbate anemia and leukopenia. Russian patients tend to access ART earlier than many of their African counterparts due to more robust diagnostic infrastructures in urban centers. However, stigmatization, sociopolitical barriers, and poor adherence in key populations (eg, people who inject drugs) can lead to inconsistent treatment, with variable hematologic outcomes. The frequency of iatrogenic cytopenias due to ART and prophylactic antibiotics (eg, cotrimoxazole, ganciclovir) is another consideration, particularly in resource-rich settings where drug availability is high^[34,35]^.

### Clinical and diagnostic implications

These ethnoracial disparities in baseline hematologic indices challenge the use of uniform cut-off values for defining cytopenias. The reliance on generalized reference ranges derived from Caucasian populations may lead to overdiagnosis of cytopenias in Africans or underestimation of severity in Russians, thereby affecting treatment decisions. For instance, a neutrophil count of 1.2 × 10^9^/L may be considered pathological in Russian cohorts but normal in African individuals with BEN. Similarly, anemia thresholds may need to be contextualized with respect to endemic nutritional deficiencies or parasitic burdens.

### Comparative trends in HIV-related cytopenias

Understanding the comparative trends in HIV-associated cytopenias among African and Russian populations unveils significant ethnogeographic disparities in disease presentation, progression, and response to treatment. These trends are influenced by a complex interplay of biological, environmental, and health systems factors that shape hematological outcomes in PLHIV (Table 1)^[11,14,36]^.

**Table 1:** A comparative table summarizing average hemoglobin, leukocyte, white blood cell differentials, and platelet counts among African and Russian HIV-infected cohorts

Parameter	African PLHIV (mean ± SD)	Russian PLHIV (mean ± SD)	Notes
Hemoglobin (g/dL)	10.5 ± 2.1	12.2 ± 1.8	Observational cohort studies, 2005–2022
Total WBC (×10^9^/L)	4.2 ± 1.5	5.1 ± 1.3	Adjusted for ART use and opportunistic infections
Neutrophils (%)	48 ± 12	55 ± 10	Includes consideration of BEN in Africans
Lymphocytes (%)	40 ± 10	35 ± 9	Lymphopenia is more prevalent in Africans with advanced HIV
Monocytes (%)	8 ± 3	7 ± 2	Limited variation; reflects innate immune activation
Eosinophils (%)	2 ± 1	2 ± 1	Rarely altered, except with parasitic coinfections
Platelet count (×10^9^/L)	175 ± 60	210 ± 55	Thrombocytopenia more common in African cohorts

### Anemia trends

Anemia remains the most prevalent cytopenia among HIV-infected individuals globally, but its occurrence, severity, and underlying causes vary between Africans and Russians. Studies indicate a markedly higher burden of anemia in sub-Saharan Africa, with prevalence estimates ranging from 60% to 80% in some cohorts. This high incidence is often linked to nutritional deficiencies, late-stage presentation, coinfections such as malaria and tuberculosis, and delayed ART initiation. In contrast, anemia in Russian PLHIV, while common, tends to occur with moderate severity and is more frequently associated with chronic inflammation, hepatitis coinfection, alcohol-induced marrow suppression, and ART-related mitochondrial toxicity. Importantly, macrocytic anemia – often a side effect of zidovudine (AZT) use – is more frequently reported in Russian patients, where ART adherence is more established, whereas microcytic or normocytic anemia predominates in African patients, typically due to iron deficiency or anemia of chronic disease. These observations highlight the need for population-specific diagnostic algorithms and therapeutic strategies to address the distinct etiologies of anemia in each group^[11,37,38]^.

### Leukopenia and neutropenia patterns

Leukopenia, especially neutropenia, demonstrates significant ethnoracial variation in HIV-infected individuals. BEN, prevalent among individuals of African descent, complicates the interpretation of neutrophil counts and may mask clinically significant neutropenia in African patients. Consequently, standard leukocyte thresholds used in Western populations may not be appropriate for African cohorts. Conversely, in Russian PLHIV, neutropenia is more often acquired, related to ART side effects, advanced disease, or co-treatment with myelosuppressive agents like ganciclovir or interferon therapy for hepatitis C. Furthermore, lymphopenia, particularly CD4+ T cell depletion, occurs universally across populations but may progress more aggressively in African patients due to higher rates of untreated or late-diagnosed infections. However, data suggest that immune recovery post-ART may be more robust in African cohorts despite lower baseline counts, potentially due to genetic and immunologic differences[36].

### Thrombocytopenia distribution

Thrombocytopenia in HIV has multifactorial origins, including immune-mediated platelet destruction, marrow suppression, and coinfections. Its prevalence varies geographically, with African cohorts exhibiting a moderate-to-high incidence, often associated with advanced HIV and HBV coinfection. In Russian populations, thrombocytopenia is frequently linked to HCV coinfection, alcohol-related liver disease, and splenomegaly. ITP-like presentations may be more common in Russians during early HIV infection, whereas in Africans, thrombocytopenia often correlates with systemic disease progression[25].

### Pancytopenia observations

Pancytopenia, though less frequent, is a critical indicator of advanced HIV or infiltrative marrow pathology such as lymphoma, disseminated tuberculosis, or hematologic malignancy. In both African and Russian populations, pancytopenia often reflects late-stage disease. However, due to more frequent delays in healthcare access and diagnosis, African patients are more likely to present with profound cytopenias and poorer prognostic indices at the time of initial evaluation^[26,39]^.

### Impact of art and coinfections

The introduction of ART has markedly reduced the incidence of severe cytopenias globally. However, the degree of hematologic normalization post-ART initiation varies. African patients, particularly those initiating ART in advanced stages, may exhibit slower hematologic recovery. Additionally, persistent infections (eg, malaria, helminths, TB) continue to contribute to cytopenias despite viral suppression. In contrast, Russian patients face cytopenic complications from hepatotropic viruses, alcohol use, and ART toxicity, which may necessitate regimen modifications[40].

### Sex- and age-related differences

Demographic variables further modify the landscape of HIV-associated cytopenias. Female patients in Africa often experience more severe anemia, potentially due to menstruation, pregnancy-related demands, and nutritional deficits. In Russia, younger individuals, especially intravenous drug users, are at greater risk for neutropenia and thrombocytopenia due to compounded immunosuppressive factors and poor ART adherence. Age-related marrow senescence may further exacerbate cytopenias in older HIV-positive populations across both regions^[41–43]^.

### Diagnostic and therapeutic implications

The recognition of ethnoracial disparities in HIV-related cytopenias among African and Russian populations has profound diagnostic and therapeutic implications. Clinicians must approach cytopenia assessment and management through the lens of population-specific hematological baselines, regional disease burdens, and healthcare system dynamics. Failure to account for these contextual factors risks underdiagnosis, overtreatment, and suboptimal patient outcomes.

### Diagnostic considerations

Standard hematological reference ranges, predominantly derived from Caucasian populations, may not be suitable for individuals of African descent. For instance, the lower baseline neutrophil counts seen in individuals with BEN could be misclassified as pathological neutropenia, potentially leading to unnecessary ART regimen changes or prophylactic interventions. Diagnostic thresholds for anemia and thrombocytopenia must also be contextualized, especially in regions where iron deficiency, helminthic infections, and micronutrient insufficiencies are endemic. In Russian populations, diagnostic precision is often hindered by comorbid conditions such as hepatitis B and C coinfections, alcohol-related bone marrow suppression, and drug abuse. These overlapping pathologies necessitate a more nuanced interpretation of cytopenias. Bone marrow biopsy, viral load monitoring, and liver function tests may be essential to differentiate HIV-related cytopenias from alternative etiologies. Moreover, in both populations, diagnostic algorithms should integrate nutritional assessments, inflammatory markers, and CD4+ T cell counts to determine the severity and potential reversibility of the hematologic abnormalities^[44–47]^.

### Therapeutic strategies

Therapeutic approaches must be tailored to the underlying cause of cytopenia while considering ART access, adherence, and toxicity profiles. In African settings, anemia management should prioritize nutritional rehabilitation, treatment of parasitic infections, and early ART initiation. The use of zidovudine (AZT), though historically common, should be minimized in severely anemic patients due to its myelotoxic effects. Iron supplementation, folate, and vitamin B12 may be essential adjuncts in populations with dietary deficiencies. For neutropenia, clinical vigilance is advised to distinguish BEN from infection- or drug-induced cases; G-CSF may be considered in severe, symptomatic cases. In Russia, where ART regimens are more accessible, management of cytopenias often involves switching to less myelosuppressive drugs or treating coinfections that contribute to marrow suppression. Antiviral therapy for hepatitis B or C, discontinuation of alcohol, and managing ART side effects play critical roles. Thrombocytopenia management may require corticosteroids or IVIG for immune-mediated cases, especially in early HIV stages. However, in both settings, consistent follow-up, patient education, and laboratory monitoring are key to successful treatment outcomes^[48–50]^.

### Mechanistic details of therapeutic toxicity pathways

ART, while lifesaving, can contribute to hematologic toxicity through several mechanistic pathways affecting bone marrow function and peripheral blood cells. Nucleoside reverse transcriptase inhibitors (NRTIs), particularly AZT, impair mitochondrial DNA synthesis in hematopoietic progenitor cells, leading to ineffective erythropoiesis and anemia. This myelosuppressive effect also reduces granulocyte and megakaryocyte proliferation, contributing to leukopenia and thrombocytopenia[48]. Non-nucleoside reverse transcriptase inhibitors (NNRTIs) and certain protease inhibitors may indirectly induce cytopenias through immune-mediated mechanisms or by exacerbating underlying nutritional deficiencies, hepatic dysfunction, or coinfections[49]. Additionally, ART can alter cytokine profiles – elevating TNF-α, IFN-γ, and IL-6 – thereby suppressing hematopoiesis and enhancing peripheral destruction of blood cells. Drug-drug interactions with co-administered medications, such as ganciclovir or chemotherapeutics, can further potentiate marrow toxicity. Recognizing these mechanistic pathways is critical for differentiating ART-induced cytopenias from HIV disease–related or coinfection–associated hematologic abnormalities, enabling clinicians to optimize therapy and minimize adverse hematologic outcomes[50].

### Global and policy-level implications

**Figure 2.**
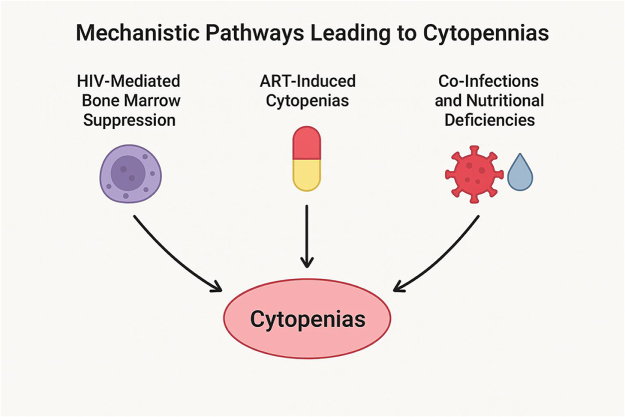
Mechanistic pathways leading to cytopenias.

From a public health perspective, the disparities in diagnostic access and therapeutic options between African and Russian PLHIV populations reflect broader inequities in healthcare infrastructure and resource allocation. There is a pressing need for localized hematological reference standards, region-specific treatment guidelines, and investment in diagnostic technologies. Training of healthcare professionals to interpret hematological variations within ethnoracial contexts can prevent mismanagement and enhance patient-centered care. Furthermore, integrating hematologic monitoring into national HIV treatment programs – especially in resource-limited African settings – can enable earlier detection of cytopenias, prevent complications, and improve survival outcomes. Multidisciplinary strategies that address the socioeconomic determinants of health, such as food insecurity and substance use, are equally important in both populations. International collaborations and research into population-specific hematologic norms and ART tolerability profiles are necessary to advance equitable HIV care (Fig. 2)^[51–53]^.

### Therapeutic approaches

The management of HIV-associated cytopenias in African and Russian populations requires a tailored therapeutic approach that addresses the unique ethnic, environmental, and socioeconomic factors influencing hematological outcomes. While the general treatment for cytopenias in HIV-infected individuals includes ART, management strategies for cytopenias such as anemia, neutropenia, and thrombocytopenia must be adjusted based on the ethnoracial differences observed in these populations. Therapeutic interventions must be individualized to account for the underlying pathophysiology of cytopenias and the presence of coinfections, comorbidities, and socioeconomic challenges^[49,50]^.

### Management of HIV-associated anemia

Anemia remains one of the most common cytopenias observed in HIV-infected individuals. In African populations, anemia is frequently complicated by coinfections such as malaria, helminthiasis, and nutritional deficiencies (e.g. iron deficiency), all of which require a multifaceted therapeutic approach. First-line treatment typically includes ART to control HIV replication and improve immune function, thereby indirectly alleviating anemia. However, ART may exacerbate anemia in some cases, particularly with drugs like AZT, which is known for its myelosuppressive effects. In such cases, switching to alternative ART regimens, such as those with integrase inhibitors or NNRTIs, may be beneficial to mitigate the impact on red blood cell production^[54–58]^. For African patients, concurrent treatment of coinfections is also critical in managing anemia. Malaria, for instance, may require prompt antimalarial therapy to treat the underlying cause, while helminthic infections necessitate deworming medications to correct iron deficiency anemia. Nutritional support, including iron and folate supplementation, is often indicated, particularly in individuals with significant dietary deficiencies. Additionally, blood transfusions may be necessary in cases of severe anemia, although this requires careful monitoring for transfusion-transmitted infections, especially in regions with limited access to blood screening technologies^[54,55]^. In Russian populations, the approach to managing anemia often involves addressing other common comorbidities, such as hepatitis or alcohol-related liver disease. These conditions can exacerbate anemia through mechanisms like impaired liver function and bone marrow suppression. ART regimens must be carefully selected to minimize myelosuppression, and dose adjustments may be required to prevent further hematological deterioration. Additionally, iron supplementation and erythropoiesis-stimulating agents may be used in patients with anemia secondary to chronic disease or ART-induced myelosuppression. Clinicians must also assess for any signs of bone marrow failure, which may necessitate further investigation and the use of growth factors like erythropoietin^[11,59,60]^.

### Management of HIV-associated neutropenia

Neutropenia in HIV-infected individuals can be the result of direct bone marrow suppression due to HIV itself or as a side effect of ART, particularly with the use of NRTIs like zidovudine. In both African and Russian populations, managing neutropenia involves balancing the suppression of HIV with the need to avoid further myelosuppression^[61,62]^. In African populations, the prevalence of BEN, a condition characterized by naturally lower neutrophil counts in individuals of African descent, must be carefully considered before initiating therapeutic interventions. In cases of mild neutropenia without clinical signs of infection, no immediate intervention is necessary. However, in individuals with severe neutropenia or recurrent infections, growth factors like G-CSF can be administered to stimulate neutrophil production. ART adjustments may also be required, particularly if the neutropenia is secondary to NRTI use, and switching to alternative regimens with a lower risk of myelosuppression can improve hematological outcomes[63]. For Russian patients, neutropenia is more likely to be associated with co-morbidities like hepatitis or alcohol use, which can cause additional bone marrow suppression. In these cases, addressing the underlying condition is critical. If neutropenia is severe or associated with frequent infections, G-CSF or other growth factors may be used to support immune function. Furthermore, for individuals with HIV and hepatitis C coinfection, antiviral therapy targeting hepatitis may help alleviate neutropenia by reducing the viral load and improving liver function, indirectly supporting the bone marrow[64].

### Management of HIV-associated thrombocytopenia

Thrombocytopenia in HIV-infected individuals can arise from immune-mediated destruction, bone marrow suppression, or coinfections such as malaria or tuberculosis. In African populations, where thrombocytopenia may be exacerbated by concurrent infectious diseases, an integrated approach to therapy is crucial. ART is the cornerstone of treatment, as viral load reduction often leads to an improvement in platelet counts. In cases of ITP, corticosteroids, IVIG, or even splenectomy in refractory cases may be considered^[65–67]^. Coinfections like malaria can exacerbate thrombocytopenia, and treating these infections is essential for improving platelet counts. For African patients, blood transfusions are often used in cases of severe thrombocytopenia, but due to concerns over blood safety, proper screening protocols should be followed. In areas with high HIV prevalence, regular screening for coinfections and prompt management of any identified conditions are critical in preventing and treating thrombocytopenia[68]. In Russian populations, thrombocytopenia is often associated with chronic liver disease or immune-mediated processes, and addressing these conditions through ART and antiviral therapy is the first step in improving platelet counts. For those with chronic hepatitis C coinfection, antiviral treatment can reduce the inflammation and fibrosis that contribute to platelet sequestration. In more severe cases of thrombocytopenia, platelet transfusions may be necessary, but careful monitoring for potential complications such as disseminated intravascular coagulation (DIC) is required. In cases of drug-induced thrombocytopenia, switching to non-myelosuppressive ART agents is recommended[69].

### Integrated approaches and personalized care

Overall, the management of HIV-associated cytopenias in African and Russian populations requires an integrated approach that addresses the multifactorial nature of cytopenias in these groups. Personalized care is essential, considering individual risk factors such as ethnicity, comorbidities, and coinfections, as well as the specific hematological variations observed in each population. The use of ART is central to treatment, but regimens must be chosen carefully to avoid exacerbating cytopenias, and coinfections should be promptly identified and treated. Supportive therapies, such as the use of growth factors, blood transfusions, and corticosteroids, must be employed judiciously, considering the risks and benefits for each patient. Furthermore, healthcare providers must remain vigilant about the potential for adverse drug reactions and interactions, particularly in populations with unique genetic profiles or high rates of comorbidities. By adopting a holistic, individualized therapeutic approach, clinicians can improve hematological outcomes and overall quality of life for HIV-infected individuals in both African and Russian populations^[70,71]^.

### Clinical implications of these variations

The ethnoracial variations observed in HIV-associated cytopenias between African and Russian populations carry significant clinical implications that impact both the diagnostic and therapeutic management of these patients. Understanding the underlying causes and prevalence rates of anemia, neutropenia, and thrombocytopenia in these populations is essential for providing optimal care, improving patient outcomes, and avoiding unnecessary treatments or misdiagnoses.

### Anemia management and differential diagnosis

Anemia is a common manifestation of HIV infection, but its pathophysiology can vary significantly between populations. In African patients, anemia is often multifactorial, with factors such as malaria, helminthic infections, and iron deficiency exacerbating the condition. These additional etiologies can complicate the diagnosis, requiring healthcare providers to consider both HIV-related causes and coinfections when assessing the severity of anemia. Clinicians must ensure that standard anemia treatments, such as iron supplementation, are appropriately prescribed, particularly in the context of infectious comorbidities. Furthermore, HIV treatment regimens may need to be adjusted to account for the impact of AZT, which is known to cause myelosuppression and exacerbate anemia. For Russian populations, where comorbidities like hepatitis B and C, alcohol-related liver disease, and bone marrow suppression due to substance use are prevalent, the management of anemia requires a more nuanced approach. The overlap between HIV and these chronic conditions necessitates regular monitoring of liver function, hemoglobin levels, and bone marrow activity. In such cases, it may be more appropriate to modify ART regimens, switch to less myelosuppressive drugs, and concurrently address the underlying conditions contributing to anemia^[71–73]^.

### Neutropenia and BEN

Neutropenia is another common hematological issue in HIV-infected patients, but the interpretation and management of low neutrophil counts are complicated by ethnoracial factors. In African populations, the prevalence of BEN, a condition characterized by lower baseline neutrophil counts, is relatively high. This physiological variation must be considered to avoid overdiagnosis and overtreatment, particularly in individuals who are asymptomatic and not at increased risk for infections. Clinicians need to recognize that a lower neutrophil count in African patients may not always indicate a pathological condition and should be distinguished from true neutropenia, which warrants intervention. In Russian patients, neutropenia may often be linked to factors such as ART-related myelosuppression, viral coinfections (e.g. hepatitis), or alcohol-related bone marrow suppression. Thus, a careful and thorough diagnostic workup is crucial to differentiate benign neutropenia from pathological neutropenia in these patients. G-CSF might be considered in cases of severe neutropenia, but only after ruling out other causes. Importantly, the management of neutropenia in Russian HIV patients may involve not only ART adjustment but also treatment of the underlying hepatic or substance-related conditions^[74–76]^.

### Thrombocytopenia and coinfections

Thrombocytopenia is another common hematological finding in HIV-infected individuals, with distinct clinical implications for African and Russian populations. In Africa, thrombocytopenia may often result from concurrent infections such as malaria, dengue, or tuberculosis, which can independently cause platelet destruction or sequestration. The management of thrombocytopenia in these cases requires careful consideration of the underlying infectious etiology and may involve interventions to treat these infections in addition to ART. Thrombocytopenia secondary to HIV infection itself typically improves with effective ART, although in severe cases, platelet transfusions may be necessary. In Russia, thrombocytopenia is frequently associated with HIV-related ITP or drug-induced thrombocytopenia, especially with older ART regimens or co-medications used for HIV-related comorbidities. Immune-mediated thrombocytopenia may require corticosteroids or IVIG for management. Additionally, in patients with chronic hepatitis coinfection, thrombocytopenia could be exacerbated by liver dysfunction and portal hypertension, necessitating an integrated approach to treatment that addresses both HIV and hepatic pathology.

### Long-term management and prognostic considerations

The clinical implications of these variations in HIV-associated cytopenias go beyond immediate management and impact long-term patient care. For both African and Russian populations, understanding the unique hematological challenges associated with HIV allows clinicians to anticipate potential complications such as infections, bleeding, and treatment resistance. By recognizing and addressing these population-specific trends in cytopenias, healthcare providers can optimize ART regimens, minimize adverse effects, and improve long-term outcomes. Moreover, the use of population-specific diagnostic criteria and treatment protocols can reduce unnecessary interventions and improve patient adherence to HIV treatment. In regions where healthcare resources are limited, as in many African countries, education and training of healthcare providers on these ethnoracial hematological trends are crucial to ensuring that patients receive appropriate care. Additionally, public health strategies aimed at preventing coinfections and improving access to ART can help reduce the burden of cytopenias and improve overall HIV care^[77–79]^.

### Population-specific manifestations of art resistance and diagnostic protocols

ART resistance presents distinct patterns across ethnoracial populations, influenced by genetic background, treatment access, and adherence challenges. In African HIV-infected cohorts, ART resistance is often compounded by delayed treatment initiation, inconsistent drug supply, and high prevalence of HIV-1 subtypes (e.g. A, C, and D), which may harbor differential susceptibility to NRTIs and NNRTIs[77]. Clinically, resistance may manifest as virologic failure accompanied by persistent or worsening cytopenias, particularly anemia and thrombocytopenia, reflecting both uncontrolled viral replication and ongoing immune dysregulation. In Russian populations, ART resistance frequently arises in the context of concentrated epidemics among people who inject drugs, exposure to multiple treatment regimens, and higher prevalence of HIV-1 subtype A/F recombinants. Here, resistance may present subtly, often detectable only via genotypic or phenotypic testing, with cytopenias less pronounced but with greater risk of hepatic and metabolic complications[78].

Population-specific diagnostic protocols must integrate these epidemiologic and genetic differences. In Africa, routine monitoring often relies on clinical indicators, hematologic parameters, and periodic viral load testing, while genotypic resistance testing remains limited in many regions[79]. In contrast, Russian guidelines incorporate more systematic viral load and resistance testing, enabling earlier detection of ART failure. Tailoring diagnostic strategies to population context – considering viral subtype, coinfections, nutritional status, and access to laboratory infrastructure – is critical to timely identification of resistance, optimizing ART regimens, and mitigating hematologic complications^[80–83]^.

### Future directions

Despite significant advances in understanding HIV-associated cytopenias, several critical knowledge gaps remain, particularly regarding ethnoracial disparities. Future research should prioritize the following areas:

1. Large-scale genomic studies: Comprehensive genomic analyses are needed to elucidate genetic determinants of cytopenias, including variants beyond Duffy-null and CXCR2 polymorphisms. Such studies could clarify population-specific susceptibility to BEN and other hematologic abnormalities[76].
2. Longitudinal assessment of ART impact: Prospective studies evaluating the long-term hematologic effects of various ART regimens are essential. These studies should stratify outcomes by ethnicity, ART regimen, coinfections, and nutritional status to better understand predictors of cytopenia development and recovery[77].
3. Interventions targeting coinfections and nutritional deficiencies: Given the high prevalence of HBV, HCV, tuberculosis, and micronutrient deficiencies in HIV-infected populations, research should explore integrated intervention strategies. These could include optimized treatment for coinfections, targeted supplementation programs, and context-specific public health strategies to mitigate cytopenias[78].
4. Population-specific clinical guidelines: There is a need for evidence-based, ethnoracially tailored guidelines for the diagnosis, monitoring, and management of cytopenias in HIV-infected populations, especially in regions with limited healthcare resources[81].
5. Mechanistic studies of hematologic toxicity: Further mechanistic research is warranted to delineate how ART, coinfections, and host genetic factors converge to influence hematopoiesis. Insights from such studies could inform safer ART regimen selection and novel therapeutic approaches.

Addressing these research gaps will enhance personalized care for HIV-infected individuals, reduce hematologic complications, and improve long-term clinical outcomes across diverse populations.

## Conclusion

HIV-associated cytopenias remain a critical clinical concern, significantly influencing disease prognosis, treatment decisions, and quality of life in affected individuals. This review underscores the ethnoracial variations in the prevalence, etiology, and clinical presentation of cytopenias among African and Russian populations living with HIV. While anemia, neutropenia, and thrombocytopenia are shared hematologic complications across geographic regions, their underlying mechanisms and associated comorbidities are shaped by distinct genetic, environmental, and socioeconomic factors. African populations, burdened by late HIV diagnosis, endemic coinfections, and nutritional deficiencies, often present with severe and multifactorial cytopenias. In contrast, Russian patients, though benefiting from a more robust healthcare infrastructure, are challenged by comorbid conditions such as hepatitis coinfections, alcohol abuse, and ART toxicity. These differences highlight the necessity for population-specific diagnostic benchmarks and individualized therapeutic strategies that address the unique hematological and systemic profiles of each group.

## Data Availability

Not applicable.
